# MFAP5 facilitates the aggressiveness of intrahepatic Cholangiocarcinoma by activating the Notch1 signaling pathway

**DOI:** 10.1186/s13046-019-1477-4

**Published:** 2019-11-27

**Authors:** Jian-Hui Li, Xiao-Xu Zhu, Fu-Xi Li, Chen-Song Huang, Xi-Tai Huang, Jie-Qin Wang, Zhuo-Xing Gao, Shi-Jin Li, Qiong-Cong Xu, Wei Zhao, Xiao-Yu Yin

**Affiliations:** 1 Present Address: Department of Pancreato-Biliary Surgery, The First Affiliated Hospital of Sun Yat-sen University, Guangzhou, 510080 China; 2 Key Laboratory of Stem Cells and Tissue Engineering (Sun Yat-Sen University), Ministry of Education, Guangzhou, 510080 China

**Keywords:** MFAP5, Intrahepatic cholangiocarcinoma, Cancer biomarkers, NOTCH, Cell cycle arrest

## Abstract

**Background:**

Intrahepatic cholangiocarcinoma (ICC) is the second most common primary liver cancer. The dismal outcome of ICC patients is due to lack of early diagnosis, the aggressive biological behavior of ICC and the lack of effective therapeutic options. Early diagnosis and prognosis of ICC by non-invasive methods would be helpful in providing valuable information and developing effective treatment strategies.

**Methods:**

Expression of microfibrillar-associated protein 5 (MFAP5) in the serum of ICC patients was detected by ELISA. Human ICC specimens were immunostained by MFAP5 antibodies. The growth rate of human ICC cell lines treated with MFAP5 or *MFAP5* shRNAs was examined by CCK8 and colony formation assays. Cell cycle analysis was performed with PI staining. The effect of MFAP5 inhibition was assessed by xenograft models in nude mice. RNA-seq and ATAC-seq analyses were used to dissect the molecular mechanism by which MFAP5 promoted ICC aggressiveness.

**Results:**

We identified MFAP5 as a biomarker for the diagnosis and prognosis of ICC. Upregulated MFAP5 is a common feature in aggressive ICC patients’ tissues. Importantly, MFAP5 level in the serum of ICC patients and healthy individuals showed significant differential expression profiles. Furthermore, we showed that MFAP5 promoted ICC cell growth and G1 to S-phase transition. Using RNA-seq expression and ATAC-seq chromatin accessibility profiling of ICC cells with suppressed MFAP5 secretion, we showed that MFAP5 regulated the expression of genes involved in the Notch1 signaling pathway. Furthermore, FLI-06, a Notch signaling inhibitor, completely abolished the MFAP5-dependent transcriptional programs.

**Conclusions:**

Raised MFAP5 serum level is useful for differentiating ICC patients from healthy individuals, and could be helpful in ICC diagnosis, prognosis and therapies.

## Background

Intrahepatic cholangiocarcinoma (ICC) is a highly aggressive and molecularly heterogeneous tumor arising from the epithelial cells of segmental or proximal branches of bile duct. ICC ranks as the second most lethal malignancies worldwide and accounts for 10–15% of all primary liver malignancies. Over the last 10–20 years, the rapidly increasing incidence and high mortality rates of ICC globally become the focus of concern among clinicians [1, 2]. Diagnosis of ICC is traditionally based on radiologic, serologic, and pathologic evaluations, but early diagnosis of ICC by non-invasive methods remains a great challenge [3]. Thus, ICC are largely diagnosed at a non-curable metastatic stage. Moreover, owing to the aggressive feature of ICC tumors, the postoperative prognosis of ICC patients is far from satisfying [4]. The recurrence rates of ICC after surgery are up to 79.5% [5]. There is urgent need to seek more effective diagnostic/prognosis biomarkers and targeted approaches for managing this challenging liver cancer.

The extracellular matrix (ECM) is a highly dynamic structure of noncellular components, including structural proteins (predominantly collagens), matricellular proteins [e.g., periostin, thrombospondins, osteopontin and secreted protein acidic and rich in cysteine (SPARC)] [6], proteoglycans, glycoproteins, and polysaccharides [7]. ECM not only provides structural and biochemical support to tumor tissue, but also remodels the tumor microenvironment leading to tumor progression acceleration and resistance to therapy. ECM molecules are also released into the bloodstream and might represent as biomarkers of tumor development. There is a growing body of evidence that hypersecretion of ECM proteins (e.g., periostin, tenascin-C) has also been associated with poor prognosis in patients following surgical resection of ICC [8]. A better understanding of how ECM remodeling affects ICC progression will contribute to the development of new diagnosis/prognosis markers and therapeutics.

Microfibrillar-associated protein 5 (MFAP5) is an ECM glycoprotein, and a component of microfibrils of the ECM that function in tissue development. MFAP5 secreted by mesenchymal stroma cells plays an essential role in hematopoiesis and immune systems. Loss function of MFAP5 inhibits bone loss in mice, whereas MFAP5 mutation is associated with the pathology of thoracic aortic aneurysms and dissections in human [9]. In addition, MFAP5 is crucial in regulating tumor progression in breast cancer, ovarian cancer and tongue cancer [10, 11]. In ovarian cancer, cancer-associated fibroblasts-derived MFAP5 upregulates lipoma-preferred partner (LPP) gene to enhance the efficacy of chemotherapy [12]. Depletion of MFAP5 by siRNA significantly decreases ovarian tumor growth and metastasis [13]. Moreover, in human cholangiocarcinoma (CCA), YAP signaling activated MFAP5 secretion to promote tube formation of human microvascular endothelial cells [14]. However, the role of MFAP5 in ICC remains unclear.

In the present study, we identify MFAP5 as a useful serum biomarker in the diagnosis of ICC. MFAP5 promotes ICC cell growth and cell cycle transition through activation NOTCH pathway. Thus, NOTCH inhibitors may represent effective regents to block MFAP5 mediated ICC cell outgrowth.

## Materials and methods

### Patients’ specimens

**Fig. 1:**
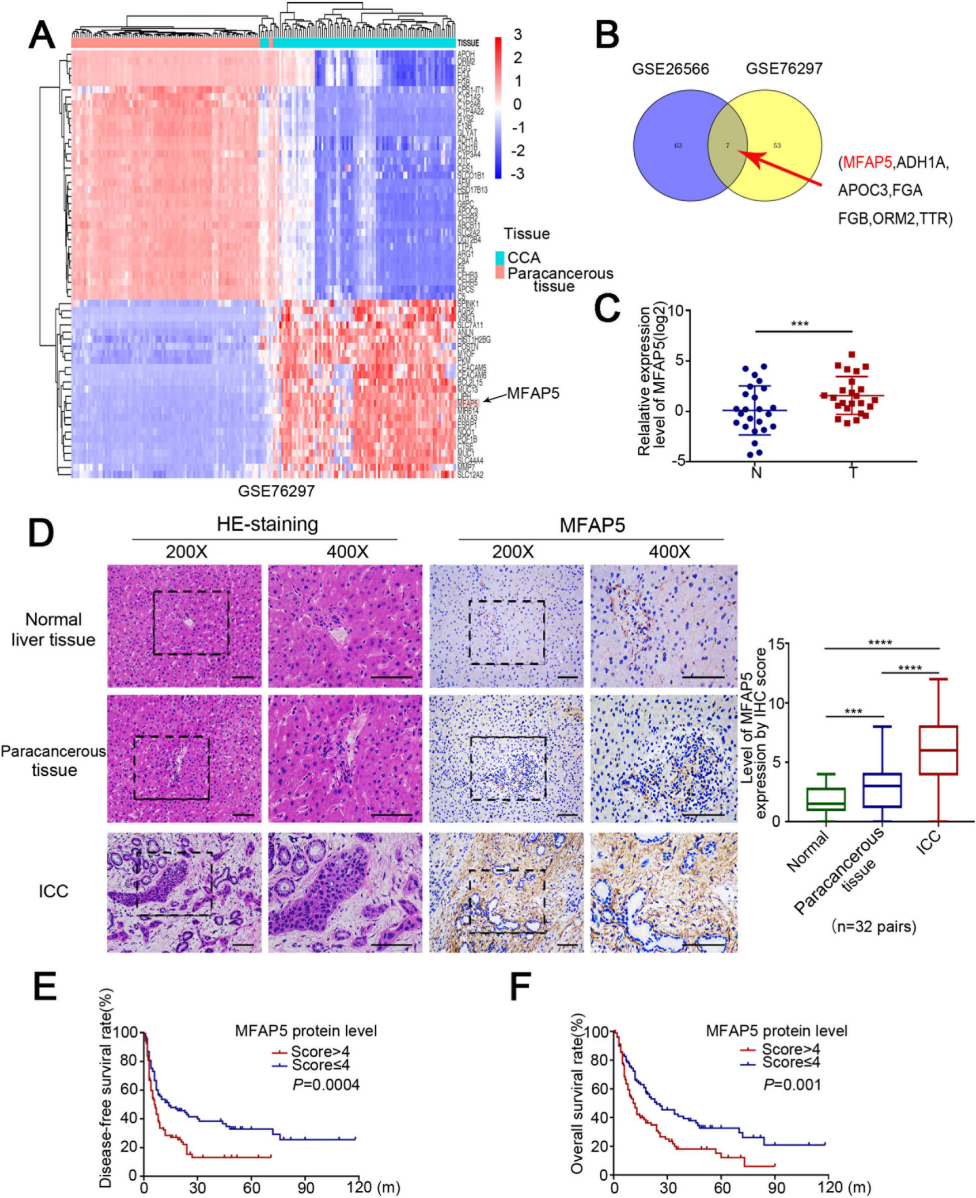
MFAP5 expression was upregulated in ICC patients and correlated with poor prognosis. **a** Heatmap showed the expression of genes in CCA tissues and non-cancerous tissue from the dataset GSE76297. **b** Venn diagram represented 7 genes that were differently expressed in the two GEO datasets. **c** Relative expression level of MFAP5 was detected by RT-QPCR. **d** Protein expression level of MFAP5 in liver tissues were detected by IHC. e, **f** Prognostic results based on MFAP5 expression in ICC tissues. *P<0.05, **P<0.01, ***P < 0.001, ****P < 0.0001

**Fig. 2:**
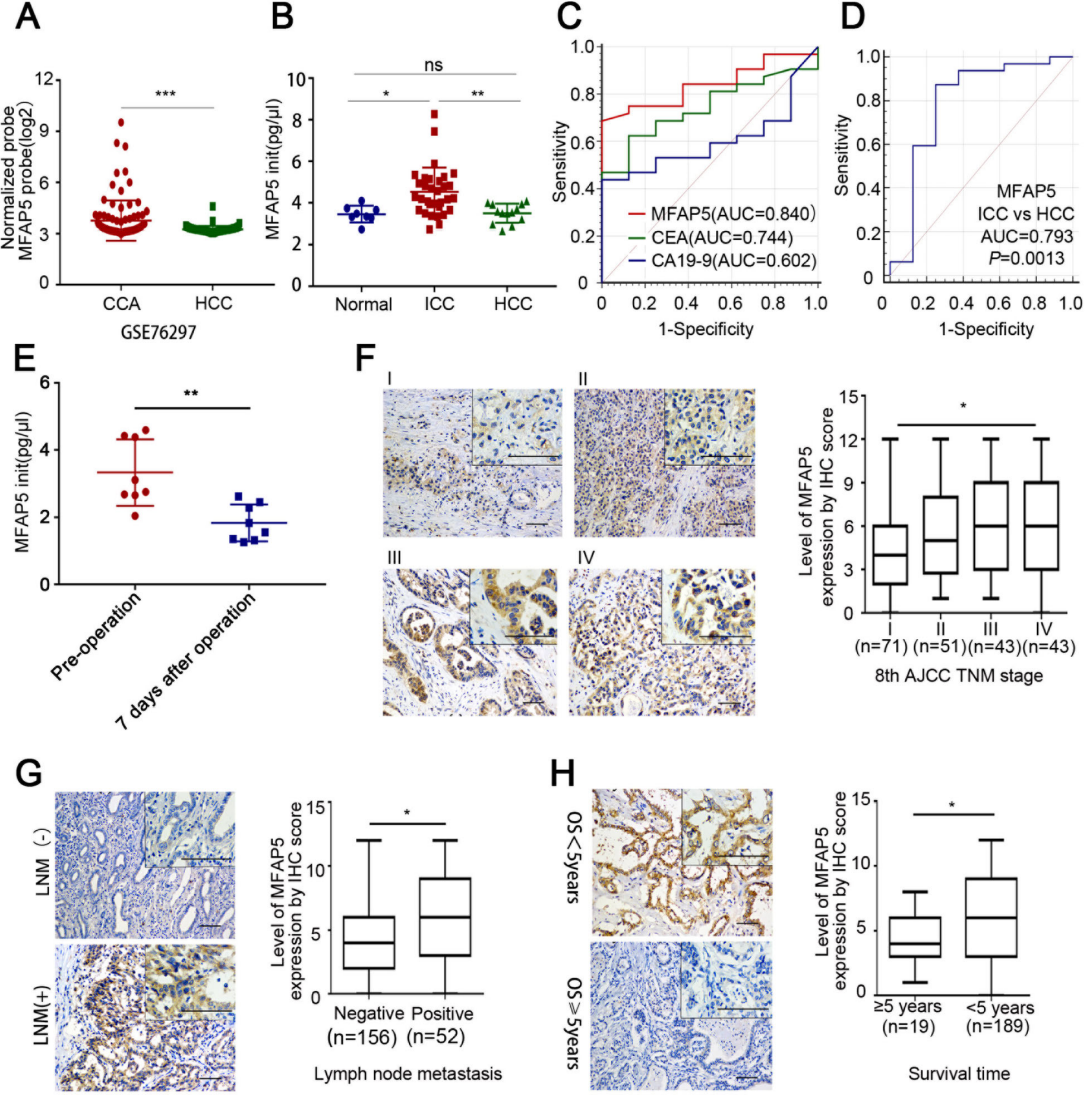
MFAP5 serum level was elevated in ICC patients**. a** MFAP5 expression level in CCA and HCC tissues from dataset GSE76297. **b** MFAP5 serum level (ELISA) in healthy volunteers, ICC patients and HCC patients (samples = 8,32,13 respectively). **c**, **d** AUC of ICC patients,healthy volunteers and HCC patients based on MFAP5 serum level. **e** MFAP5 serum level (ELISA) of pre-operation and 7 days after operation in 8 ICC patients. **f**, **g**, **h** IHC results and box plots of MFAP5 protein level in ICC tissues grouped by ICC TNM stages **f**, lymph node metastasis **g** and five-year overall survival **h**. Scale bar = 50 μm *P<0.05, **P<0.01, ***P < 0.001

240 ICC patients, who had been diagnosed by histology and underwent radical hepatectomy at the First Affiliated Hospital of Sun Yat-sen University in Guangzhou China, were included in this study. The inclusion criteria were as follows: [1] histologically diagnosed ICC [2]; underwent radical hepatectomy [3]; survived longer than 30 days after hepatectomy [4]; had integrated clinicopathological data and follow-up data. Patients who met one of the following criteria were excluded from the study: [1] diagnosed perihilar cholangiocarcinoma (Klatskin tumor) [2]; with tumors mixed of HCC and ICC [3]; R1 or R2 resection or laparotomy with tumor biopsy [4]; had received neoadjuvant chemotherapy or radiotherapy before hepatectomy. 208 ICC patients underwent hepatectomy from January 2007 to June 2016 were recruited for immunohistochemistry assay and prognostic analysis. 40 ICC patients who underwent hepatectomy from September 2016 to October 2019 were recruited for immunohistochemistry assay, qRT-PCR assay and ELISA assay (32 patients were recruited for immunohistochemistry assay in Fig. 1d, qRT-PCR assay in Fig. 1c and ELISA assay in Fig. 2b; another 8 patients were recruited for ELISA assay in Fig. 2e). 8 healthy people and 13 hepatocellular carcinoma (HCC) patients were recruited for ELISA assay (Fig. 2b). Clinicopathological characteristics of ICC patients were shown in Additional file 1: Table S1. This study was approved by the Ethical Committee of the First Affiliated Hospital of Sun Yat-sen University [15].

### Extraction and processing of gene expression omnibus (GEO)

One Affymetrix Human Transcriptome Array [HTA-2_0] dataset (GSE76297) and one Illumina human Ref-8 v2.0 expression dataset (GSE26566) were selected. The raw data was downloaded from GEO database (https://www.ncbi.nlm.nih.gov/gds/). The array data of GSE76297 included 92 CCA tissue samples and 92 non-cancerous tissue samples. The dataset of GSE26566 consisted of 103 CCA tissue samples and 59 non-cancerous tissue samples. The specific analysis method had been described previously [16].

### Immunohistochemical (IHC) staining

This staining assay was performed as described previously [17]. Slides containing the sections were stained with commercially available anti-MFAP5 (1:100,#ab283028,Abcam) and anti-Ki-67 (1:100, #ab156956,Abcam) antibodies. Staining intensity (negative, 0; mild, 1; moderate, 2; severe, 3) and proportion of positive cells (negative,0; ≤10%, 1; > 10 and ≤ 33%, 2; > 33 and ≤ 66%, 3; > 66%, 4) were quantified respectively. Two experienced pathologists scored the stained tissues independently.

### Enzyme-linked immunosorbent assay (ELISA)

The expression level of MFAP5 in ICC patients’serum was evaluated using ELISA kit (SEF590Hu Cloud-Clone Corp). The collected serum was all derived from preoperative clinical diagnosis of ICC patients.

### Cell culture and transfection

Human ICC cell lines RBE and SSP-25 were purchased from the Cell Resources Center of Shanghai Institutes for Biological Science, Chinese Academy of Science (Shanghai, China). Cells were cultured in RPMI-1640 (Gibco BRL, Rockville, MD, USA) and supplemented with 10% fetal bovine serum (Gibco BRL). For the gene knockdown assays, cells were infected with lentivirus encoding shRNA respectively. The target sequences used for MFAP5 shRNAs and Control shRNAs were listed in Additional file 1: Table S2.

### Western blot analysis

Western blot analysis was performed as described previously [18]. The anti-MFAP5 antibody (#ab283028) and anti-Ki67(#ab156956) antibody were purchased from Abcam. The anti-β-actin (**#**4970), anti-rabbit IgG, HRP-linked antibody (#7071) and Notch activated targets antibody sample kit (#68309) were obtained from Cell Signaling Technology. The anti-CCND1 (60186–1-Ig), anti-CDK4 (11026–1-AP), anti-CDK6 (14052–1-AP), anti-CDKN1A (10355–1-AP) and anti-CDC25A (55031–1-AP) antibodies were purchased from Maygene Co. The anti-NOTCH2(cleaved Ala1734) (#PA5–37433) antibody was purchased from Thermo Fisher Scientific.

### Co-immunoprecipitation (co-IP)

2 × 10^7^ RBE and SSP-25 PBS-washed cells were harvested and lysed with Pierce lysis buffer (Thermo, cat#8990) containing protease inhibitors cocktail (TargetMol, cat#C0001). Then incubated with NOTCH1 (CST,cat#3608), MFAP5 (abcam,ab203828) antibody and control IgG (CST,cat#3900) after centrifugation respectively. Subsequently, the cell lysates were incubated with Protein A/G Dynabeads (Thermo, cat#10002D/10004D). Afterwards, the protein A/G Dynabeads were eluted and collected. The eluent was boiled and denatured for Western-Bolt.

### Assay for transposase accessible chromatin with high-throughput sequencing

Chromatin preparation Nuclei was prepared from 4 × 10^4^ cells. Library amplification was performed using the NEBnext High Fidelity 2× PCR Master Mix (#M0541S, New England Biolabs) according to previously published PCR conditions. ATAC-seq library preparations were sequenced using single-end 50-bp reads on the Illumina HiSeq 2000 platform. Raw reads were adaptor-trimmed using Trim Galore (v0.2.5) and aligned to the genome with Bowtie (v1.0.1) with the m1 option enabled to allow only uniquely aligned high-quality reads. Peaks were called using the MACS2 software (v2.1.0.20140616) with the options −q 0.05 to retain significant peaks and shift size 50 to account for the transposase fingerprint, while default parameters were used for other options.

### Statistical analysis

Statistical analyses were carried out by SPSS software 24.0 and GraphPad Prism 7.0 (La Jolla, CA, USA). Results were presented as mean ± standard deviation (SD). Student t-test (paired/unpaired) was used for values following normal distribution. Non-parametric data were compared using the Mann-Whitney U test and Wilcoxon test. Chi-Square test was used for testing the differences between categorical variables. Fisher’s exact test was used when the number of variables was lower than 5. Correlation analysis were performed using the Spearman correlation tests. The survival curves were obtained by Kaplan–Meier method and compared using the log-rank test. The optimal cut-off value of continuous variables was determined by ROC curve analysis. The DeLong test was used to compare the difference between the AUCs of different biomarkers [19]. The ANOVA test was performed to compare the mean values of proliferation rate in different groups. It was considered to be statistically different when *P* < 0.05 at two-tail (*P < 0.05, **P < 0.01, ***P < 0.001, ****P < 0.0001).

## Results

### MFAP5 expression was upregulated in ICC patients and correlated with poor prognosis

To determine the functional and clinical relevance of ECM-related genes in ICC, we first analyzed all the differentially expressed protein-coding genes within two microarray datasets (GSE76297 and GSE26566) from the GEO database. Among the seven different probes that showed consistent results in the two datasets, MFAP5 was the most significantly upregulated ECM gene in both datasets (Figs. 1a, b, Additional file 1: Figure S1a). The expression level of MFAP5 differed statistically between tumor and para-tumor tissues (Additional file 1: Figure S1b**,** c). To validate these findings, we evaluated the expression level of MFAP5 in a cohort of 24 pairs of ICC and para-tumor (non-cancerous) tissues. The results showed that MFAP5 mRNA expression level was significantly higher in ICC tissues than in para-tumor tissues (Fig. 1c). IHC analysis showed that positive staining of the MFAP5 protein was enriched in ICC tissues, but was rarely observed in para-tumor and normal tissues (Fig. 1d). The results also showed that MFAP5 expression was significantly higher in ICC tissues than in non-cancerous tissues. Furthermore, to investigate the effect of MFAP5 expression on postoperative survival in ICC patients, we utilized univariate statistical methods to analyze clinical data from 208 ICC patients. The univariate and multivariate analysis showed that MFAP5 protein level, tumor vascular invasion, lymph node metastasis, and tumor differentiation were prognostic factors for disease-free survival (DFS) and overall survival (OS) (Table 1). Patients were divided into two groups based on the optimal level of MFAP5, namely, high-expression group (MFAP5 > 4, *n* = 107) and low-expression group (MFAP5 ≤ 4, *n* = 101). Both of OS and DFS were significantly different between the two groups (Fig. 1e, f). These results suggested that the *MFAP5* gene might play an important role in ICC progression.

**Table 1:** Prognostic factor for DFS and OS of patients with intrahepatic cholangiocarcinoma determined by using univariate

Variable	Disease-free survival			Overall survival		
	HR	95%CI	*P*-value	HR	95%CI	*P*-value
Univariate						
Age(>58 or ≤ 58)	0.95	0.68–1.33	0.571	1.01	0.72–1.41	0.945
Gender (female/male)	1.23	0.88–1.72	0.201	1.23	0.88–1.72	0.208
Hepatitis B (−/+)	0.94	0.62–1.43	0.788	0.88	0.58–1.32	0.547
Alb>40 g/L or ≤ 40 g/L	0.88	0.63–1.23	0.455	0.85	0.61–1.18	0.329
Hepatitis C (+/−)	1.75	0.54–5.62	0.192	1.75	0.54–5.64	0.199
MFAP5 protein level(>4/≤4)^a^	1.76	1.25–2.47	0.0004	1.71	1.22–2.40	0.001
CA19–9(>37 U/L or ≤ 37 U/L)	1.70	1.22–2.38	0.031	1.91	1.37–2.66	0.043
CEA(>5μg/L or ≤ 5μg/L)	1.76	1.21–2.55	0.011	1.84	1.27–2.67	0.017
Vascular invasion (+/−)	2.32	1.36–3.95	0.0001	2.54	1.46–4.41	0.0001
Number of tumor (multiple/single)	1.79	1.19–2.68	0.0006	1.85	1.23–2.78	0.0003
Lymph node metastasis (+/−)	1.48	1.03–2.12	0.017	1.40	0.98–1.99	0.047
Adjacent organ invasion (+/−)	1.21	0.82–1.78	0.284	1.29	0.87–1.91	0.160
Tumor differentiation						
Well vs Moderately	0.44	0.22–0.91	0.095	0.38	0.19–0.74	0.041
Well vs Poorly	0.30	0.15–0.59	0.009	0.27	0.14–0.51	0.002
Moderately vs Poorly	0.60	0.41–0.88	0.002	0.61	0.42–0.90	0.005
Multivariate						
^a^MFAP5 protein level(>4/≤4)	1.88	1.29–2.75	0.0008	1.75	1.20–2.56	0.003
Vascular invasion (+/−)	1.72	1.12–2.65	0.012	1.77	1.15–2.79	0.008
Lymph node metastasis (+/−)	1.93	1.29–2.87	0.001	1.55	1.04–2.32	0.028
Tumor differentiation (Well vs Moderately vs Poorly)	1.55	1.25–1.93	0.0001	1.38	1.12–1.69	0.002

*MFAP5* Microfibril associated protein 5, *CA19–9* Carbohydrate antigen 19–9, *CEA* Carcino-embryonic antigen, *DFS* Disease-free survival, *OS*, Overall survival, *HR* Hazard ratio, *CI* Confidence interval

^a^Immunohistochemical (IHC) score, split at median

### MFAP5 serum level was elevated in ICC patients

Analysis of the GSE76297 dataset showed that there was a significant difference in MFAP5 expression between CCA and HCC patients (Fig. 2a). To test whether MFAP5 could be used as an early diagnostic serum index to discriminate ICC from HCC, we performed an exploratory analysis of MFAP5 serum level in a cohort of 32 ICC patients and 13 HCC patients. For the control, we measured MFAP5 serum level in healthy volunteers who had healthy medical reports. Analysis in this exploratory cohort revealed significantly elevated MFAP5 level in ICC patients’ serum samples compared to serum samples from healthy volunteers. Importantly, ICC patients also showed significantly higher serum MFAP5 level compared to HCC patients (Fig. 2b). Based on the elevated MFAP5 expression level in serum samples from the cohort of ICC patients, we next evaluated the diagnostic power of serum MFAP5 as a diagnostic marker for ICC by performing ROC curve analysis. The analysis revealed an AUC of 0.840 for the differentiation between healthy volunteers and ICC patients based on their initial MFAP5 serum level. The diagnostic power of initial serum MFAP5 was superior to initial CEA and CA19–9 serum level, which showed an AUC of 0.744 and 0.602 respectively (Fig. 2c). Arguing for a specific elevation of serum MFAP5 level between ICC and HCC patients, the ROC curve analysis revealed an AUC of 0.793 for the differentiation of HCC and ICC patients (Fig. 2d). To test whether MFAP5 could be used as a biomarker for ICC therapies, we performed an analysis of MFAP5 serum level in a cohort of 8 ICC patients. Each case included one sample of pre-operation and one sample of 7 days after operation. We tested MFAP5 serum level by ELISA and analyzed the data with the Paired-Sample T Test. The results showed that MFAP5 serum level was significantly higher in preoperative serum than in postoperative serum (*P* = 0.0031; Fig. 2e). This indicated that MFAP5 might be used as a biomarker for evaluating the efficiency of therapies of ICC.

To further investigate the role of MFAP5 in the aggressive progression of ICC, we explored the correlation of MFAP5 expression and the clinicopathologic characteristics of 208 ICC patients. As shown in Table 2, MFAP5 level in higher ICC TNM stages were higher than those in low ICC TNM stages (Sample numbers of stages I, II, III, IV were 71, 51, 43, 43; *P* = 0.0141; Fig. 2f), indicating a correlation between MFAP5 expression and ICC TNM stages. MFAP5 level was significantly higher in ICC cases exhibiting positive lymph node metastasis (LNM) than in those without LNM (Sample numbers of Negative and Positive LNM were 156 and 52; *P* = 0.0189; Fig. 2g). Similarly, patients survived more than five years after surgery had significantly lower MFAP5 IHC scores than patients who died within five years (Sample numbers of > = 5 years and < 5 years OS were 19 and 189; *P* = 0.0274; Fig. 2h).

**Table 2:** Correlation between MFAP5 expression and intrahepatic cholangiocarcinoma in 208 ICC patients

Characteristics	Number of patients		P-value^a^
	High MFAP5 expression	Low MFAP5 expression	
Gender			1.000
Female	49	46	
Male	58	55	
Age			0.581
>58	53	46	
≤ 58	54	55	
Tumor			0.7815
>5 cm	55	49	
≤ 5 cm	52	52	
Tumor number			0.473
single	69	77	
Multiple	38	34	
Tumor differentiation			0.477
Well	4^b^	5	
Moderate	64	67	
Poor-undifferentiated	39	29	
Vascular invasion			0.720
Not present	86	84	
Present	21	17	
Lymph node invasion			0.0001
Not present	67	89	
Present	40	12	
AJCC 8th TNM stage			0.006
I	35	31	
II	17	35	
III	27	16	
IV	28	15	
3-year survival			0.058
≥ 36 months	16	26	
<36 months	91	75	
5-year survival rate			0.030
≥ 60 months	5	14	
<60 months	102	87	

^a^Chi-square test

^b^Fisher’s exact test

### MFAP5 promoted ICC cells proliferation both in vitro and in vivo

**Fig. 3:**
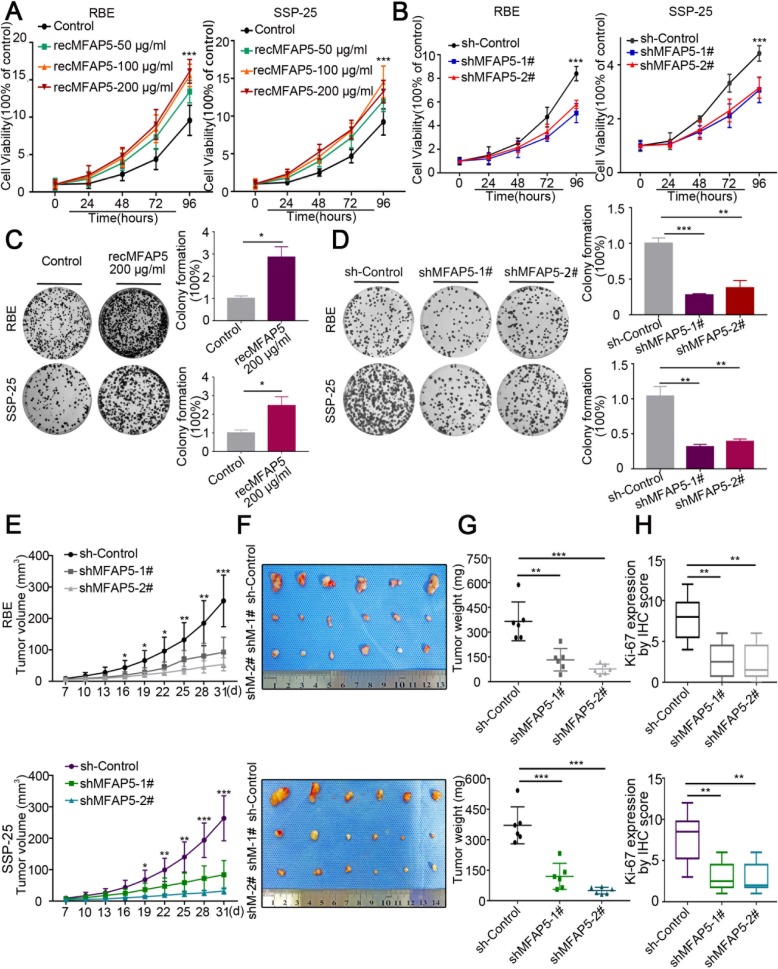
MFAP5 promoted proliferation of ICC cells in vitro and in vivo**. a**, **b** Cell viability results showed the different proliferation rate after co-cultured with recMFAP5 and after transfected MFAP5 shRNAs. **c**, **d** Colony formation assay results showed the different colony numbers in co-cultured experiments of recMFAP5 and transfected MFAP5 shRNAs cells. **e** Tumor growth curves after injected ICC cells. **f** Xenograft tumors from respective groups were shown. **g** Tumor weight of different xenograft tumors groups. **h** Boxplot showed Ki-67 level in sh-MFAP5 and sh-Control derived xenograft tumors which evaluated by IHC scores. *P < 0.05,**P < 0.01,***P<0.001

To investigate the function of MFAP5 in the progression of ICC, RBE and SSP-25 human ICC cell lines were co-cultured with purified recombinant MFAP5 protein (recMFAP5). The results demonstrated that exogenous recMFAP5 increased ICC cells’ proliferation in a dose-dependent manner (Fig. 3a). We also established stable MFAP5 knockdown RBE and SSP-25 ICC cells with two respective *MFAP5* shRNAs (Additional file 1: Figure S2a, b). The proliferation rate was significantly inhibited in MFAP5 knockdown RBE and SSP-25 cells compared to control cells (Fig. 3b). Moreover, colony-forming ability was markedly promoted by recMFAP5 in both RBE and SSP-25 cells (Fig. 3c). In contrast, down-regulation of MFAP5 substantially suppressed colony formation in RBE and SSP-25 cells (Fig. 3d).

To evaluate whether the expression level of MFAP5 could affect ICC cell growth in vivo, we subcutaneously injected MFAP5 knockdown or control RBE and SSP-25 cells into nude mice. We observed that the tumor growth rate of MFAP5-silenced groups was markedly slower than that of the control groups for both RBE and SSP-25 cell lines (Fig. 3e). The mice were euthanized, and the subcutaneous tumors were measured every 3 days until 31 days after cell injection (Fig. 3f). The tumor weight was significantly lower in the MFAP5-silenced groups than in the control groups (Fig. 3g). Furthermore, IHC staining revealed that the expression of MFAP5 and Ki-67 were markedly downregulated in MFAP5 knockdown tumors (Figs. 3h, Additional file 1: Figure S2c–f).

### MFAP5 facilitated CCND1/CDK4/6/CDC25A-dependent G0/G1 to S-phase transition

**Fig. 4:**
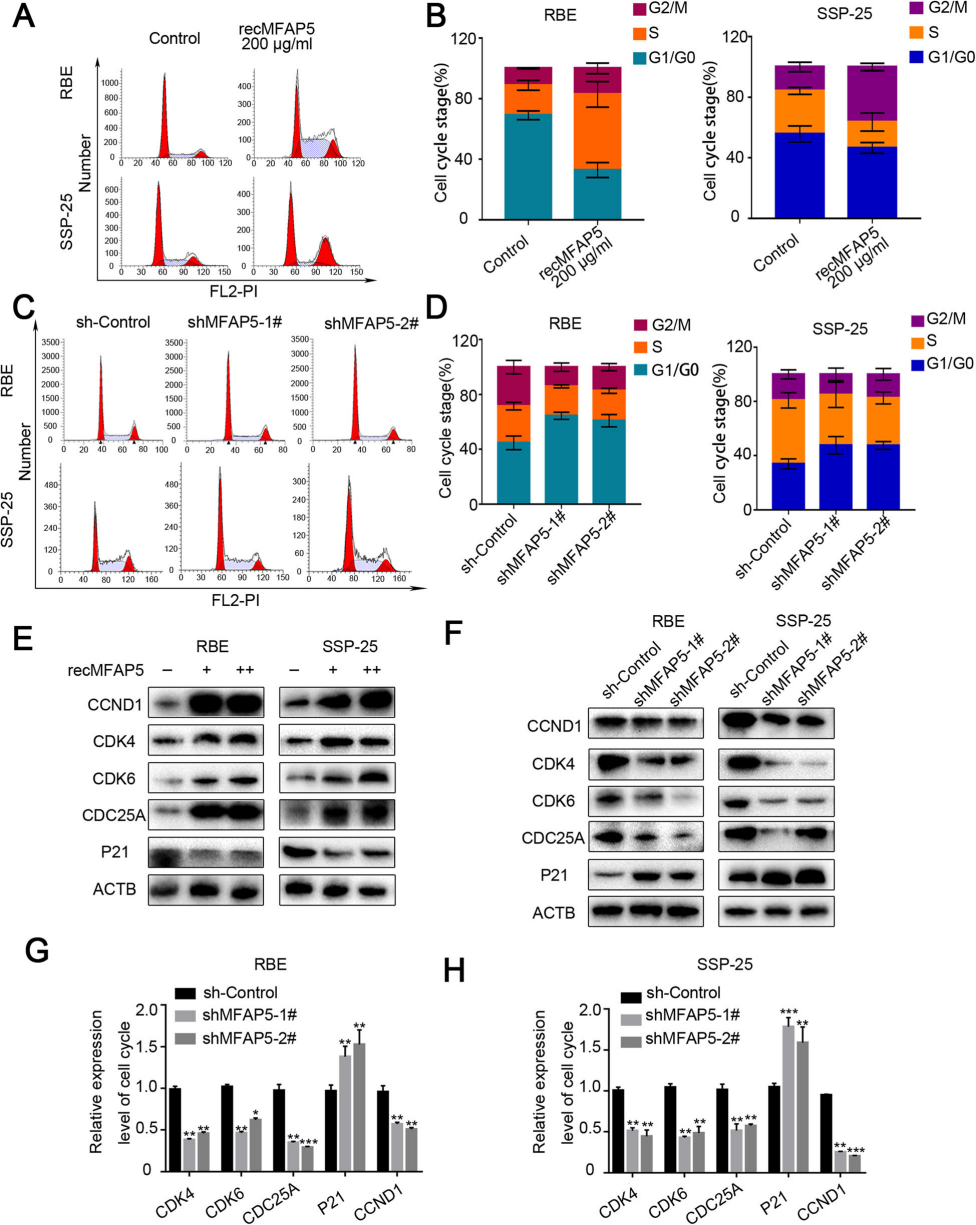
MFAP5 facilitated ICC cell cycle transition. **a**, c Cell cycle flow cytometry analysis showed the different G0/G1 percentage after co-cultured with recMFAP5 and after transfected sh-MFAP5. **b**, **d** Three replicate experiments on cell cycle analysis. **e** Western blot assay showed the expression level of CCND1, CDK4, CDK6 CDC25A, P21 in ICC cells line after co-cultured with recMFAP5 and after transfected sh-MFAP5. **g**, **h** RT-PCR results showed the relative expression level of cell cycle genes in sh-MFAP5 ICC cell lines. *P < 0.05,**P < 0.01,***P<0.001

To explore whether tumor growth promoted by MFAP5 is due to cell cycle acceleration, cell cycle analysis was performed after treating REB and SSP-25 cells with recMFAP5. Treatment with recMFAP5 decreased the fraction of cells in the G0/G1 phase and increased the fraction of cells in G2/M compared to the DMSO control (Fig. 4a, b). Addition of MFAP5 resulted in increased numbers of RBE cells in the S phase, but did not increase the proportion of S-phase SSP-25 cells; this may have been due to MFAP5-induced S-phase SSP-25 cells entering the G2/M phase. Similarly, MFAP5 silencing resulted in fewer cells in G2/M and an increase in the fraction of cells in the G0/G1 phase (Fig. 4c, d). However, the percentages of cells in the S phase were not lower with MFAP5 knockdown. These results indicate that the ICC cells could be arrested at the G0/G1 phase via silencing of MFAP5.

We also investigated the mechanisms underlying the effects of MFAP5 on the G0/G1 to S-phase transition. Given that CDK4/6/CDC25A are important for the G0/G1 to S transition during the cell cycle, we postulated that CDK4/6/CDC25A may participate in MFAP5-mediated cell cycle regulation. This hypothesis was supported by the data showing that recMFAP5 treatment increased CCND1/CDK4/6/CDC25A expression but reduced p21 expression in ICC cells (Fig. 4e). In addition, CCND1/CDK4/6/CDC25A protein and mRNA levels were significantly attenuated by MFAP5 knockdown, whereas p21 expression was increased (Fig. 4f, g, h). These results suggest that MFAP5 may increase CCND1/CDK4/6/CDC25A transcription by inhibiting p21 activity and thus promote G0/G1 to S-phase transition and cell proliferation.

### MFAP5 positively regulated the transcription of Notch1 pathway genes

**Fig. 5:**
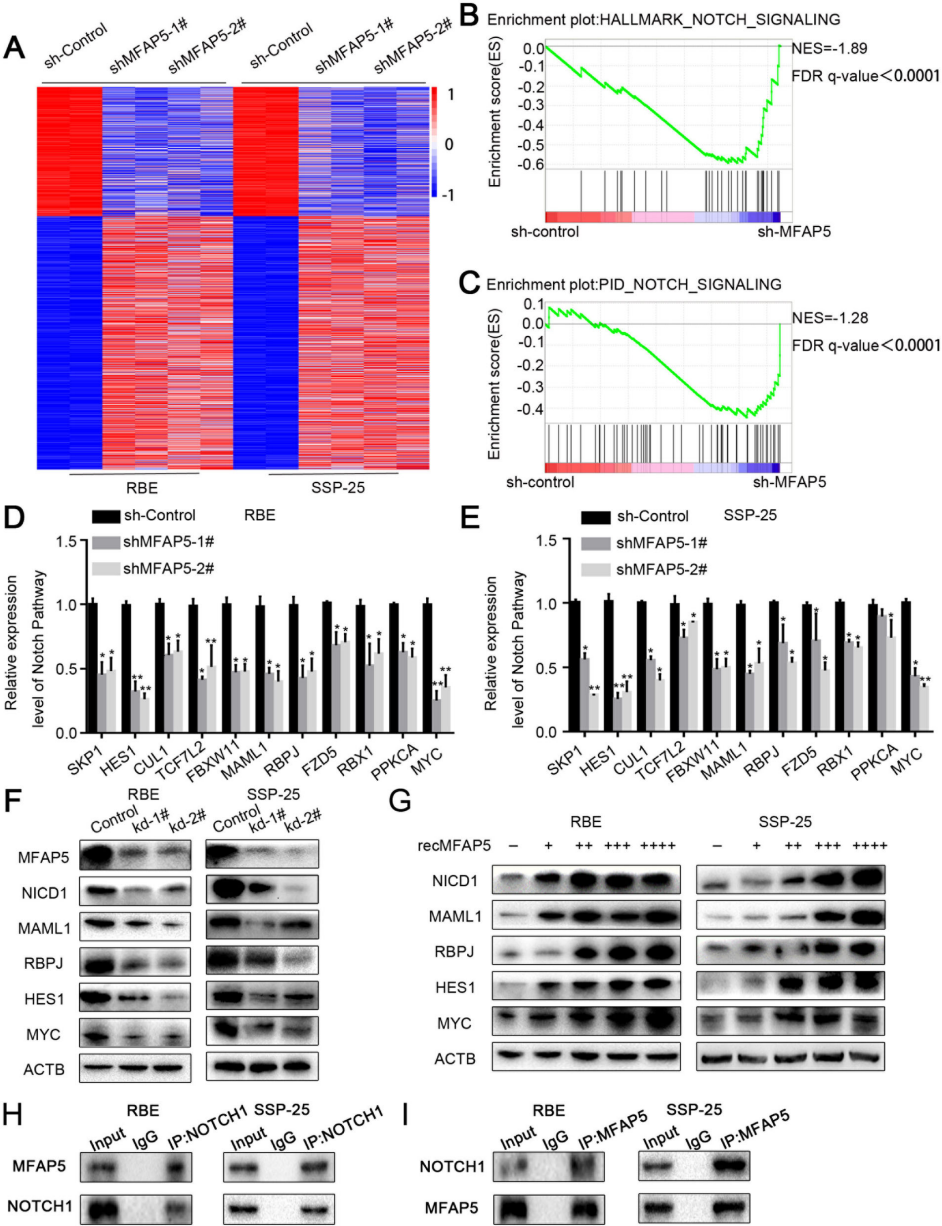
MFAP5 positively regulated Notch1 pathway transcription. **a** Heat map showed results of RNA-seq of SSP-25 and RBE transformed sh-control, sh-MFAP5–1# and sh-MFAP5–2#. **b**, **c** GSEA results showed down-regulation of Notch pathway in cells transfected sh-MFAP5 compared with sh-Control. **d**, **e** RT-PCR results showed the mRNA level of genes in NOTCH1 pathway after transfected sh-MFAP5 in ICC cell lines. **f**, **g** Western blot analysis of Notch1 pathway target genes in sh-MFAP5 and sh-Control cells (**f**) and co-culture with recMFAP5 and control cells (G, each “+” represented 50μg/ml recMFAP5). **h**, **i** Co-IP results showed that MFAP5 could bind directly with NOTCH1. *P < 0.05,**P < 0.01,***P<0.001

To further characterize the regulatory effect of MFAP5 on the cell cycle and cell proliferation, the transcription status of ICC cells transduced by MFAP5 shRNA or control shRNA (shControl) was compared using RNA-seq (Figs. 5a, Additional file 1: Figure S3a, 3b). The gene set enrichment analysis (GSEA) showed that the mRNA levels of Notch signaling pathway genes were markedly reduced with MFAP5 silencing (Fig. 5b, c). We next validated the regulatory effect of MFAP5 on components of the Notch signaling pathway. Western blot and qPCR analysis showed that several Notch signaling pathway components, as well as Notch signaling targets, were reduced in MFAP5 knockdown cells compared to shControl cells (Fig. 5d, e). Additionally, shMFAP5 treatment resulted in significant repression of the NOTCH1 intracellular domain (NICD1), MAML1, and HES1, but there was no significant difference in NOTCH2 intracellular domain (NICD2) (Additional file 1: Figure S3c, d). These results suggest that MFAP5 may interact directly with the NOTCH1 receptor to activate the Notch1 signaling pathway. To further confirm the regulatory role of MFAP5 on Notch1 signaling, the expression of these Notch1 signaling factors was compared between recMFAP5- and DMSO-treated cells using western blotting. Strong positive correlations were observed between recMFAP5 dosage and the expression of Notch1 signaling factors (Figs. 5f, g, Additional file 1: Figure S3e). These findings demonstrated that MFAP5 plays important roles in regulating the Notch1 signaling pathway. In order to explore how MFAP5 regulated Notch1 pathway, we performed the Co-IP experiment in ICC cell lines (RBE and SSP-25). The results showed that MFAP5 could bind directly with NOTCH1, indicating that MFAP5 regulated Notch1 pathway by interacting directly with Notch1 receptor (Fig. 5h, i).

### Chromatin accessibility by ATAC-seq analysis revealed a role for MFAP5 in Notch1 activation

**Fig. 6:**
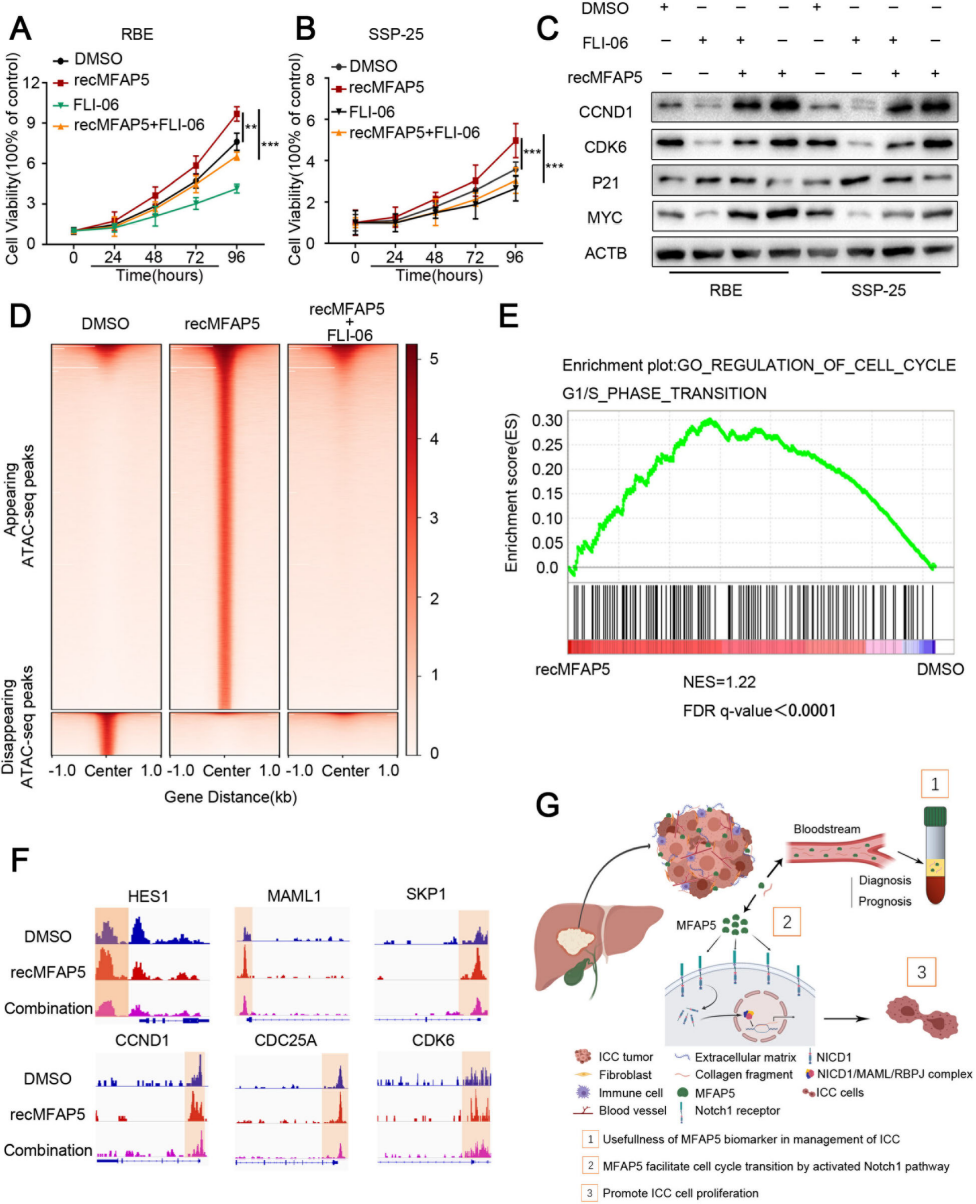
Chromatin Accessibility by ATAC-seq Analysis Reveals a Role of MFAP5 for NOTCH1 Activation. **a**, **b** CCK-8 assay showed the proliferation differences of ICC cells co-cultured with recMFAP5 and combination (recMFAP5 + FLI-06). **c** Western blot results of cell cycle genes in ICC cell lines after co-cultured with recMFAP5 and FLI-06. **d** ATAC-seq heatmap results showed the appearing peaks status in different groups. **e** GSEA analysis revealed genes in the G1/S phase following recMFAP5 co-culture. **f** The gene peaks results revealed changes in different groups in RBE cell line. **g** Proposed schematic models illustrated MFAP5 facilited the aggressiveness of ICC via modulating Notch pathway/G1S signaling axis

As the Notch1 signaling pathway was shown to be involved in MFAP5-mediated ICC aggressiveness, we next investigated the effect of the NOTCH inhibitor FLI-06 on recMFAP5-induced cell outgrowth. Interestingly, the accelerated proliferation of ICC cells induced by MFAP5 was significantly inhibited in the presence of FLI-06 (Fig. 6a, b). Further analysis revealed that FLI-06 could effectively abolish the MFAP5-induced overexpression of CCND1, CDK6, CDKN1A, and MYC (Fig. 6c). These findings indicate that the Notch1 pathway is important for MFAP5-enhanced ICC cell growth.

To further identify the target factors regulated by the MFAP5/Notch1 axis, genome-wide chromatin accessibility was assessed by Assay for Transposase-Accessible Chromatin using sequencing (ATAC-seq). There were significant changes in chromatin architecture with appearing peaks associated with upregulated gene expression in recMFAP5-treated ICCs compared to the DMSO control (Fig. 6d). Surprisingly, many appearing peaks in recMFAP5-treated ICCs were absent in ICCs co-treated with recMFAP5 and FLI-06. We interpret this finding to mean that FLI-06 suppressed the downstream transcription events induced by MFAP5/Notch1 signaling. The results of the GSEA analysis showed an enrichment of genes assigned to the cell cycle gene ontology (GO) term that were inactivated in FLI-06-treated ICCs and significantly upregulated in ICCs treated with recMFAP5 (Fig. 6e). Figure 6f shows representative examples of these target genes, including *CCND1*, *CDC25A*, and *CDK6*. The results also revealed significant changes in chromatin architecture, with disappearing peaks associated with FLI-06 co-culture.

## Discussion

The ECM regulates tissue homeostasis, and ECM dysregulation contributes to tumor progression. The ECM factors within an abnormally remodeled ECM can enhance ICC tumor progression and aggressiveness. We demonstrated here that MFAP5 is highly expressed in the ECM of ICC and is correlated with worse outcome. MFAP5 secreted by ICC tumors enters the blood of patients and can be detected specifically and sensitively by ELISA experiments. MFAP5 in the ECM enhanced the activation of the Notch1 pathway, facilitating downstream gene transcription and thereby promoting G1 to S cell cycle phase transition as well as proliferation (Fig. 6g). Given the lack of effective early ICC diagnosis methods and the difficulty in distinguishing ICC from HCC in the early stages of the diseases, our results demonstrated that MFAP5 serological detection may be used as an effective method for early ICC diagnosis and for evaluating the efficiency of therapies; in turn, MFAP5 inhibition would likely be used as a treatment. Our findings also revealed an important mechanism underlying amplified Notch activation in ICC that is mediated by MFAP5 in the tumor microenvironment.

Identification of specific ICC diagnostic or prognostic biomarkers is urgently required. To date, few ideal screening analyses have been developed. Analysis of the CA19–9 biomarker may aid in CCA diagnosis, but the levels of CA19–9 in ICC patients are heterogeneous. Li et al. defined a biliary vesicle miR-based panel that can be used for CCA diagnosis [20], while Andresen et al. identified DNA methylation of CDO1, CNRIP1, SEPT9, and VIM displaying frequencies of 45–77% in biliary brushes from CCA patients [21]. Anti-glycoprotein 2 (anti-GP2) has also been reported as a potential diagnostic biomarker in CCA as well as in secondary sclerosing cholangitis (SSC) [22]. However, none of these biomarkers can effectively distinguish HCC from ICC, two diseases that require different surgical and postoperative drug treatments [4]. Serum metabolites have also been used as diagnostic biomarkers for CCA and HCC. These findings indicated integration of genomics, transcriptomics, and metabolomics for the identification of HCC and ICC subtypes. In our study, MFAP5 levels did not increase in the serum of either healthy volunteers or HCC patients, but increased specifically in the serum of ICC patients. The specificity and sensitivity of MFAP5 are higher than those of traditional biomarkers (e.g., CEA and CA19–9). We also found that the serum level of MFAP5 was significantly decreased in the serum of post-operation compared with the serum of pre-operation. This indicated that MFAP5 might be used as a biomarker for evaluating the efficiency of therapies of ICC. Importantly, in this study, MFAP5 was correlated with various malignant indexes (e.g., metastasis and poor OS) and can therefore be used as a prognostic marker in patients with ICC.

The cancer microenvironment contains both cellular and non-cellular components, including the extracellular matrix, and is critical for the activation of tumor survival signals. Although Notch signaling has previously been reported to regulate liver metabolism, inflammation, and cancer, the interaction between the composition in an ECM context and Notch signaling remain largely unknown [23]. A recent study has identified the interaction between the ECM and integrin α5 as the extracellular cue that activates Notch signaling in pancreatic progenitors via the F-actin-YAP1-Notch axis [24]. Notch signaling promotes ECM remodeling during endocardial projection formation, providing new insights into the pathology of congenital heart disease [25]. In our study, we showed that a NOTCH1 inhibitor phenocopied MFAP5 knockdown and abolished MFAP5-induced ICC outgrowth, strongly suggesting that MFAP5 acts upstream of Notch signaling in ICC. NICD1 levels increased in a dose-dependent manner in ICC cells treated with MFAP5 and Co-IP results showed that MFAP5 could interact directly with Notch1 receptor, verifying that MFAP5 acts upstream of Notch1 signaling in ICC. Activated Notch1 signaling elicited increased transcription of downstream genes that are reported to promote ECM remodeling in ICC. Further context-specific understanding of the MFAP5/Notch1 signaling axis in ICC will be essential to translate these findings into clinical practice.

Cell-cycle regulation by intracellular molecular pathways has been extensively studied. Additionally, the cell cycle is also regulated via cell-cell physical forces and cell-ECM interfaces [26]. Early studies established that cell shape, polarity, and adhesion strongly influence DNA synthesis and cell growth. However, substantially less is known about the mechanical regulation of the cell cycle by the ECM. Previous studies showed that MFAP5 inhibition induces G2/M phase arrest, decreases the expression of Cyclin B1, Cyclin D1, and CDK4, and enhances p21 and p53 levels in cervical cancer. Inhibition of cell growth by MFAP5 knockdown is dependent on reactive oxygen species (ROS) production [27]. In our study, we showed that MFAP5 promotes ICC G0/G1 to S-phase cell cycle transition which is dependent on Notch1 signaling activation. The Notch1 signaling pathway was reported to enhance the expression of the cyclin E protein in cholangiocellular carcinomas [28]. However, we did not observe any change in cyclin E expression, either with overexpression or with knockdown of MFAP5 (data not shown). In contrast, the levels of CCND1 and CDK4/6, regulators of the G0/S checkpoint, were significantly upregulated though MFAP5/Notch1 activation. In agreement with this, significant changes in chromatin architecture with appearing peaks were associated with upregulated *CCND1* and *CDK6* gene expression.

## Conclusions

In conclusion, our results unveil serum MFAP5 as a potential diagnostic, prognostic and therapeutic tool for ICC, providing a novel approach for use in noninvasive screening of ICC. ECM-derived MFAP5 functions as an oncogenic protein that may participate in Notch1 signaling activation in ICC. The mode and mechanism of the suppression of tumor aggressiveness by MFAP5 depletion are potentially important. Consequently, large-scale studies should be undertaken to evaluate the therapeutic potential of MFAP5 in ICC.

## Supplementary information

**Additional file 1: Figure S1**. MFAP5 expression was upregulated in cholangiocarcinoma (CCA) patients by analysis GSE26566 and GSE 76297 datasets. Related to Fig. 1. **Figure S2.** Depletion of MFAP5 secretion inhibits ICC tumor growth in vivo. Related to Fig. 3. **Figure S3.** Silencing MFAP5 suppresses the expression of HES1 and MYC in ICC cells. Related to Fig. 5. **Table S1.** The clinic pathological characteristics of 208 ICC patients. **Table S2.** Sequences of primers and shRNAs used in this study.

## Data Availability

All data generated or analyzed during this study are included in this published article and its supplementary information files.

## Abbreviations

ATAC-seq: Assay for Transposase-Accessible Chromatin using sequencing
CA19–9: Carbohydrate Antigen 19–9
CCA: Cholangiocarcinoma
CEA: Carcino-Embryonic Antigen
DFS: Disease-Free Survival
ECM: Extracellular Matrix
ICC: Intrahepatic Cholangiocarcinoma
MFAP5: Microfibril Associated Protein 5
OS: OS overall survival
RNA-seq: Whole Transcriptome Shotgun sequencing
