# Supplementary material for: MFAP5 facilitates the aggressiveness of intrahepatic Cholangiocarcinoma by activating the Notch1 signaling pathway
Source: J Exp Clin Cancer Res. 2019 Nov 27;38:476. doi: 10.1186/s13046-019-1477-4 (PMC6882185; doi:10.1186/s13046-019-1477-4)
Supplement: Supplementary file 1 — Additional file 1: Figure S1. MFAP5 expression was upregulated in cholangiocarcinoma (CCA) patients by analysis GSE26566 and GSE 76297 datasets. Related to Fig. 1. Figure S2. Depletion of MFAP5 secretion inhibits ICC tumor growth in vivo. Related to Fig. 3. Figure S3. Silencing MFAP5 suppresses the expression of HES1 and MYC in ICC cells. Related to Fig. 5. Table S1. The clinic pathological characteristics of 208 ICC patients. Table S2. Sequences of primers and shRNAs used in this study. [file 13046_2019_1477_MOESM1_ESM.docx]

**Supplementary Information**

**MFAP5 Facilitates the Aggressiveness of Intrahepatic Cholangiocarcinoma by Activating the Notch1 Signaling Pathway**

Jian-Hui Li^#1^, Xiao-Xu Zhu^#1^, Fu-Xi Li^2^, Chen-Song Huang^1^, Xi-Tai Huang^1^, Jie-Qin Wang^1^, Zhuo-Xing Gao^2^, Shi-Jin Li^1^, Qiong-Cong Xu^1^, Wei Zhao^2^*, Xiao-Yu Yin^1^*

**Contents**

**Supplementary Figure 1**

**Supplementary Figure 2**

**Supplementary Figure 3**

**Supplementary Table1**

**Supplementary Table2**

**Supplementary Methods**

**Supplementary Figure 1**

**Supplementary Figure 1. MFAP5 expression was upregulated in cholangiocarcinoma (CCA) patients by analysis GSE26566 and GSE 76297 datasets. Related to Figure 1.**

**
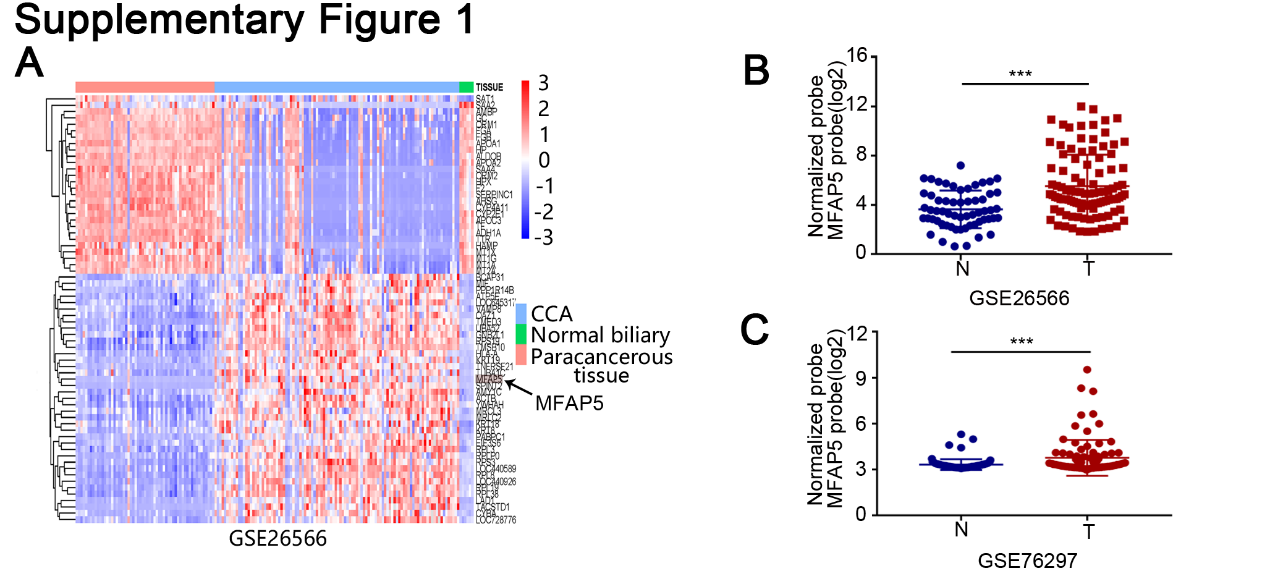
**

(A) Heatmap showed the expression of genes in CCA cancer tissue and non-cancerous tissue from the dataset GSE26566. (B) MFAP5 in the gene expression profile from dataset GSE26566. （N=59,T=103） (C) MFAP5 in the gene expression profile from dataset GSE76297. （N=92, T=92）(****P*＜0.001)

**Supplementary Figure 2**

**
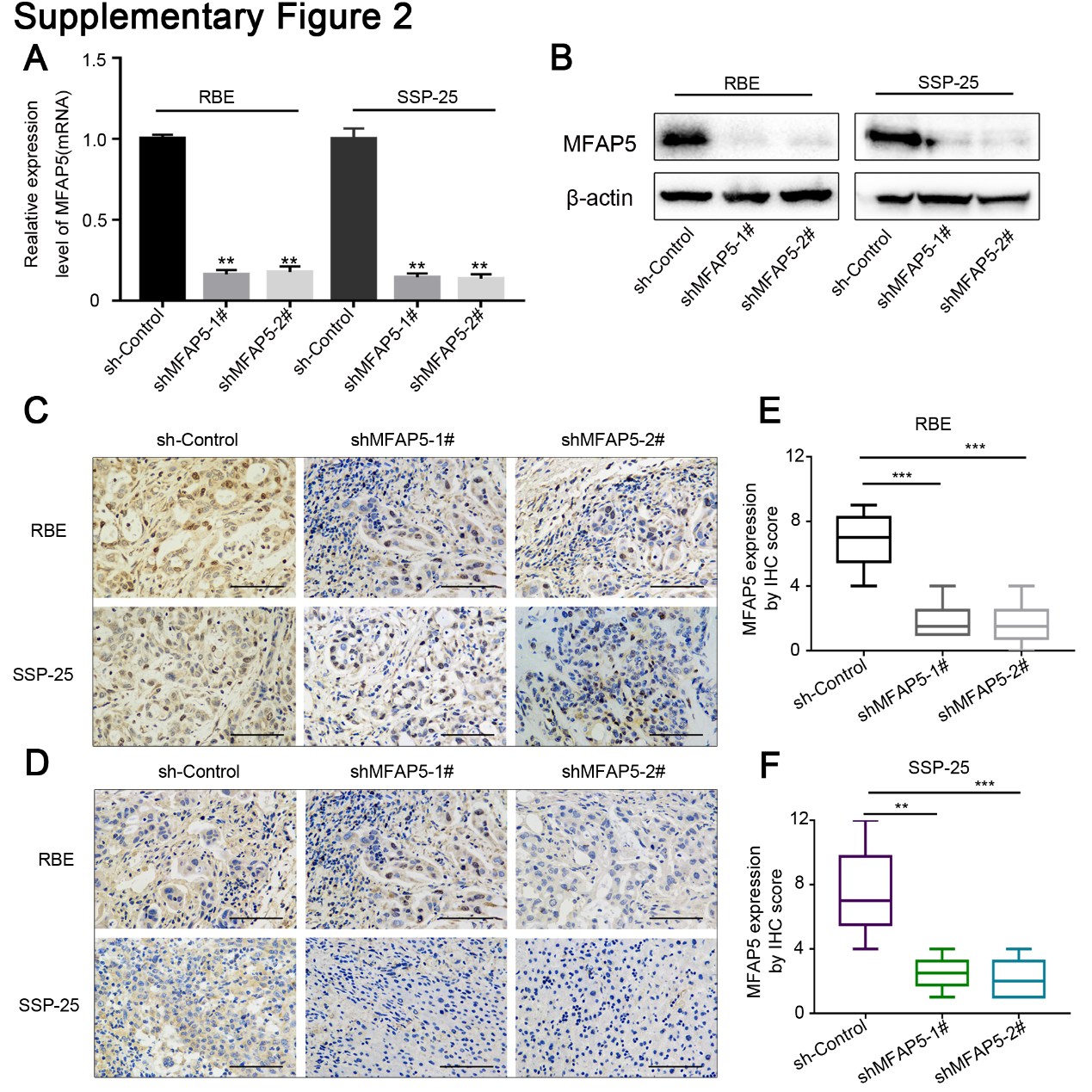
**

**Supplementary Figure 2. Depletion of MFAP5 secretion inhibits ICC tumor growth in vivo. Related to Figure 3.**

(A, B) Lower expression of MFAP5 mRNA (A) and protein (B) in RBE and SSP-25 cells after transfected sh-MFAP5 shRNA. (C, D) IHC staining results showing Ki-67 and MFAP5 positive cells in sh-MFAP5 and sh-Control derived xenograft tumors. (E and F) Boxplot showing MFAP5 positive cells in sh-MFAP5 and sh-Control derived xenograft tumors. (Scale bar=50μm ***p* < 0.01, ****p* < 0.001)

**Supplementary Figure 3**

**

**

**Supplementary Figure 3. Silencing MFAP5 suppresses the expression of HES1 and MYC in ICC cells. Related to Figure 5.**

(A) Correlation plots comparing gene expression in two MFAP5 shRNA infected RBE and SSP-25 cells by log ratio of normalized counts relative to control shRNA. R represents correlation value. (B) Volcano plot showed the distribution of differentially up-regulated (yellow) and down-regulated (blue) genes upon knock down MFAP5. HES1 and MYC were indicated with arrow. (C, D) Western blot analysis of NOTCH2 intracellular domain (NICD2) antibody in sh-MFAP5 and shControl cells (C) and co-culture with recMFAP5 and control cells (D, each “+” represented 50ug/ml recMFAP5). (E) Gray value analysis showed the expression of Notch1 pathway activated Genes and recMFAP5 was positively in linear correlation, R square were shown.

**Supplementary Table 1. The clinic pathological characteristics of 208 ICC patients**

| Characteristics | Values |
| --- | --- |
| Age, year (mean ± SD) | 58.4 ±10.5 (21-79) |
| Gender (male/female) | 113/95 |
| Tumor size, cm (≤5/＞5) | 104/104 |
| Tumor number(single/multiple) | 146/72 |
| CA19-9, kU/L (≤37/＞37) | 80/128 |
| TNM stage (I/II/III/IV)  Lymphatic metastasis (Negative, Positive) | 71/51/43/43  156/52 |
| Hepatitis B (Negative, Positive) | 165/43 |
| Vascular invasion (Negative, Positive) | 170/38 |
| Tumor recurrence, months (mean ± SD) | 15.84 ±22.44 (1-118) |
| Overall survival, months (mean ± SD) | 21.38 ± 22.43 (1-118) |

**Supplementary Table 2. Sequences of primers and shRNAs used in this study**

| Names | Sequences |
| --- | --- |
| MFAP5-shRNA-1# | GGACCCAAGGTGCTGCTGTTT |
| MFAP5-shRNA-2# | CGTATGTACATCGTCAACAAG |
| Control-shRNA | GCTTCGCGCCGTAGTCTTA |
| GAPDH-primer-F | GAGTCCACTGGCGTCTTCAC |
| GAPDH-primer-R | ATCTTGAGGCTGTTGTCATACTTCT |
| MFAP5-primer-F | GGGTCAATAGTCAACGAGGAGA |
| MFAP5-primer-R | CTGTAGCGGGATCATTCACCA |
| CCND1 -primer-F | GCTGCGAAGTGGAAACCATC |
| CCND1 -primer-R | CCTCCTTCTGCACACATTTGAA |
| CDK4-primer-F | ATGGCTACCTCTCGATATGAGC |
| CDK4-primer-R | CATTGGGGACTCTCACACTCT |
| CDK6-primer-F | GCTGACCAGCAGTACGAATG |
| CDK6-primer-R | GCACACATCAAACAACCTGACC |
| CDC25A-primer-F | GTGAAGGCGCTATTTGGCG |
| CDC25A-primer-R | TGGTTGCTCATAATCACTGCC |
| CDKN1A-primer-F | TGTCCGTCAGAACCCATGC |
| CDKN1A-prime-R | AAAGTCGAAGTTCCATCGCTC |
| HES1-primer-F | TCAACACGACACCGGATAAAC |
| HES1-primer-R | GCCGCGAGCTATCTTTCTTCA |
| MYC-primer-F | GTCAAGAGGCGAACACACAAC |
| MYC-primer-R | TTGGACGGACAGGATGTATGC |
| MAML1-primer-F | CCCCAGTGAGTCATTTCCTCT |
| MAML1-primer-R | AGGAAATGACTCACTGGGGTTA |
| CUL1-primer-F | GATCTGGGACGACCTCAGAG |
| CUL1-primer-R | CCCCTTTTTCGACTTAGAAGGAG |
| RBPJ-primer-F | CGGCCTCCACCTAAACGAC |
| RBPJ-primer-R | TCCATCCACTGCCCATAAGAT |
| SKP1-primer-F | GACCATGTTGGAAGATTTGGGA |
| SKP1-primer-R | TGCACCACTGAATGACCTTTT |
| TCF7L2-primer-F | AGAAACGAATCAAAACAGCTCCT |
| TCF7L2-primer-R | CGGGATTTGTCTCGGAAACTT |
| FBXW11-primer-F | GGAACATCATCTGTGATCGTCTC |
| FBXW11-primer-R | TGGTAAAGCGGTAATAAAGTCCC |
| RBX1-primer-F | TTGTGGTTGATAACTGTGCCAT |
| RBX1-primer-R | GACGCCTGGTTAGCTTGACAT |
| PPKCA-primer-F | GTCCACAAGAGGTGCCATGAA |
| PPKCA-primer-R | AAGGTGGGGCTTCCGTAAGT |
| FZD5-primer-F | CATGCCCAACCAGTTCAACC |
| FZD5-primer-R | CGGCGAGCATTGGATCTCC |

**Supplementary Methods**

**Quantitative real-time PCR (qRT-PCR)**

The total RNA was extracted using TRIzol reagent (Life Technologies, USA) according to the manufacturer’s instructions. The reverse transcription was performed using Maxima® First Strand cDNA synthesis kit for RT-PCR (Thermo ScientificTM, USA). qRT-PCR was performed on a Life QuantStudio 6 Flex Real-time PCR system using Takana SYBR® Primix Ex TaqTM Kit (Takana, Dalian, China). All the primers used in the experiments were shown in the **Supplementary Table 2**.

**Cell viability and a plate clone formation assay**

Cells were seeded into 96-well plates at the density of 2 x 10^3^ cells per well. Cell proliferation was detected at 24, 48, 72, and 96 h after respective treatments with Cell Counting Kit-8 (CCK8) (Dojindo, Kumamoto, Japan). Absorbance values at 450 nm (A450) were recorded as representation of cell viability.

For the plate clone formation assay, 400 cells per well were seeded in a 6-well plate and cultured for 2 or 3 weeks (2 weeks only for SSP-25 in recMFAP5/control subgroup). The culture medium was changed every 4 days. Then, the cells were fixed in 4% formaldehyde for 20 min and then stained with 1% crystal violet for 30 min. Plates were washed by PBS twice and then took pictures of these plates and calculated colony numbers with Image J.

**Cell cycle detection**

RBE and SSP-25 cells were digested and collected by 1,500 rpm centrifugation for 5 mins. The cells were rinsed with PBS and re-suspended in 1ml DNA Staining solution and 10 μl Permeabilization. The solution was mixed and incubated in the dark for 30 mins. Cell cycle was analyzed via flow cytometry.

**ICC xenograft model**

BALB/c-nude mice (4-6week-old) were purchased from Guangdong Medical Laboratory Animal Center. All experimental procedures were Model Animal Research Center of Nanjing University. 1 × 10^7^ RBE or SSP-25 cells in 150 µL PBS were inoculated subcutaneously into the left flank of nude mice. Tumors was measured every three days. Tumor volume was calculated with the formula: V= 0.5 × length × width^2^. The tumor-bearing mice were killed 33 days after inoculation, and the tumors were subsequently removed for further study.

**Gene Set Enrichment Analysis (GSEA)**

Gene Set Enrichment Analysis was supported by the Broad Institute website (http://www.broadinstitute.org/gsea/index.jsp). All GSEA analyses presented in our study were performed using the Java GSEA implementation. Alignment background references with Molecular Signatures Database v6.2.
